# Industrial symbiosis platforms for synergy identification and their most important data points: a systematic review

**DOI:** 10.12688/openreseurope.13893.1

**Published:** 2021-08-31

**Authors:** Chrysanthi Akrivou, Lucyna Łȩkawska-Andrinopoulou, Georgios Tsimiklis, Angelos Amditis

**Affiliations:** 1 Institute of Communication and Computer Systems (ICCS), National Technical University of Athens, 9, Iroon Politechniou Str., Zografou Campus, Athens, 15773, Greece

**Keywords:** Industrial Symbiosis, ICT tools, Digital platform, Data points

## Abstract

**Background**: Industrial symbiosis (IS) primarily involves interfirm utilization of industrial residual resources. An important factor determining the success of IS is the identification and matching of cooperation opportunities. Digital tools, including IS platforms, are considered facilitators of this process. This systematic literature review addresses the research question: ‘ *Which are the most important data points of an IS platform and* *how can they be used for the promotion of IS?*’.

**Methods**: The review is based on scientific publications from the following academic research databases: ScienceDirect, Scopus, SpringerLink, Wiley Online Library, AISel and IEEE (via Google Scholar), and grey literature obtained through a customized Google search technique, last performed on 9/3/2021. Records were included according to their scientific content, namely if the document: i) examined the identification of synergies utilizing ICT tools, ii) data requirements or platform related information were presented or iii) the impact of a digital tool in promoting IS was discussed. Exclusion criteria were: articles not written in English, not peer-reviewed, published before 2016 or document type other than scientific article, conference paper or EU project deliverable. Two independent reviewers performed title scanning and abstract reading of the documents to reduce the risk of bias.

**Results:** The total number of records included after abstract and full text reading was 32. The main results of this review suggest that two significant types of data points are encountered in IS platforms; i) data required for synergy identification and ii) platform related information.

**Conclusions:** A possible limitation of the study is a minor risk of bias due to one reviewer performing full text reading and synthesis of results; however, they reported to and consulted with the supervising reviewer. Overall, the results indicate that several types of data points are required for effective matching and successful promotion of IS through digital tools.

## Introduction

In recent years, interest in environmentally sustainable industrial and business models has increased, as society’s demand for resources has accelerated and related environmental problems (water scarcity, raw materials’ depletion and waste accumulation) have emerged. Industrial symbiosis (IS) is a sustainable, resource efficient and eco-friendly industrial waste management approach with long-term perspective ( Kosmol & Leyh, 2019; Lawal *et al.*, 2021; Marconi *et al.*, 2018). Initially, the term ‘industrial symbiosis’ referred to the physical exchange of material (waste, stock, by-product), energy, and/or water between geographically close companies. This exchange involves a company’s output residual flow being used by another company as input flow, so both gain profit and reduce their environmental impact. Presently, IS applies to all interfirm cooperation models, consisting of either exchange or sharing of underutilized resources, utilities, infrastructure, and services in order to achieve a green, waste-free supply chain ( Fraccascia & Yazan, 2018; Kosmol, 2019; Kosmol & Leyh, 2020).

An IS network, i.e. a network of companies entailed in synergistic relationships, can be realized via three main different pathways: planned (top-down), facilitated (intermediary) and self-organized (bottom-up). The top-down approach appertains to planned actions by managers, coordinating the involved stakeholders. Typically, eco-industrial parks (EIPs) are considered a top-down effort, either concerning the planning of new industrial complexes (greenfields) or the retrofitting of pre-existing ones (brownfields). The facilitated approach involves third parties, referred to as intermediaries, which facilitate the initiation process by supporting the exchange of essential information and knowledge. The self-organized approach results from spontaneous collaborations between stakeholders, primarily motivated by economic incentives ( Fraccascia & Yazan, 2018; Kosmol & Leyh, 2019; Lawal *et al.*, 2021; van Capelleveen *et al.*, 2018b). The size of an IS network varies in terms of geographic scope from local, to regional, national or international. A local IS network refers to the case of EIPs, where co-located industrial actors collaborate and seek to collectively achieve benefits, whereas a regional scale network refers to the cooperation of companies in a wider geographic span. A national level IS network refers to the symbiotic relationships created between companies located in the same country, and international level refers to any joint effort and partnership, physical or virtual, formed between members located across the globe ( Kosmol & Leyh, 2020; Romero *et al.*, 2017; Yeo *et al.*, 2019b; Zhang *et al.*, 2017).

One of the main barriers impeding the achievement of IS actions is the informational gap. Lack of a systematic exchange of available information prevents entities from being aware of the latent collaboration opportunities, regardless of the geographic proximity between them. However, information and communication technology (ICT) tools have been developed that are capable of overcoming this challenge, particularly to support the identification of possible business engagements. These tools have been recognized as facilitators of IS because their role is to utilize information to promote constructive synergistic relationships. Specifically, according to van Capelleveen *et al.* (2018a), six different types of information systems assisting IS have been identified, namely: open online waste markets, facilitated synergy identification systems, industry sector identification systems, social network platforms, IS knowledge repositories and region identification systems for IS. An open online waste market, or e-marketplace, enables transactions of industrial residues by matching businesses’ supply and demand streams. Facilitated synergy identification systems are slightly different to e-marketplaces, in the notion that these systems are utilized by intermediaries of an IS initiative in a more organized and systematic way. Industry sector identification systems have been developed in order to enhance the partnership identification procedure as a part of a holistic approach, by suggesting connections between industrial clusters, not between actors. Another type of information system proposed in the literature to serve as a facilitator of IS is social network platforms and social network communities, since they can support both information exchange and relationship formation. IS knowledge repositories are systems that do not allow the matching of stakeholders but rather serve in a supporting role, by providing diverse information of previous IS case studies and facilitating the discovery of potential IS opportunities. They might be in the form of a database, where users are able to query on a waste of interest, or in the form of a Wiki, based on users’ involvement for data collection. Region identification systems have been developed to assist the detection of districts with abundant possibilities of IS implementation in terms of industrial and economic activity ( Fraccascia & Yazan, 2018; Kosmol & Leyh, 2019; van Capelleveen *et al.*, 2018a).

The term platform is used without a precise definition and it is regularly used to refer to one or a combination of the aforementioned types of information systems. The structure of the platform and the tools it encompasses varies according to the type of IS network coordination, the level of intermediaries’ involvement and the geographic extent it is designed to facilitate. However, common components, features and functionalities have been recognized in the majority of the digital tools dedicated to IS. Aside from the initial identification of potential partnerships, i.e. the pursuit of compatible resources to be exchanged, a platform is proposed to further assist IS by actively promoting it. Some approaches to support succeeding tasks of the IS involve the evaluation and recommendation of potential synergies, as a means of assisting the decision-making of participants. Also, coordinating and supporting the management of transactions between actors is a complicated process that is essential for the fruition of synergies, a process that the digital tool is expected to facilitate as well. Even though the current literature discusses the digital tools facilitating IS, a thorough examination and classification of requirements and capabilities according to the type of information system is missing ( Kosmol & Leyh, 2020; van Capelleveen *et al.*, 2018a; van Capelleveen *et al.*, 2018b; Yeo *et al.*, 2019b).

Consequently, it is evident that there is a need for investigation of the elements of an IS platform affecting not only the matchmaking process, but also the supplementary support it provides to the involved members of an IS network, such as the decision-making process. The objective of this research is to investigate the key data points that affect the identification of symbiotic partners and the implementation of synergies through an IS platform, as discussed in the existing literature, and how these elements are utilized in order to efficiently promote IS through a web-based tool. The purpose is to capture and document practical information and present it in a coherent form. All type of actors interested in IS and its advancement, such as potential stakeholders, research organizations, coordinating bodies and policy makers, are expected to benefit from this research. 

## Methods

To obtain a comprehensive overview of the industrial symbiosis platforms, this study adopts the systematic literature review (SLR) approach, based on the systematic reviews’ outline provided by Harris *et al.* (2014) in medical sciences, adapted to the field of information technology, in compliance with the PRISMA guidelines ( Akrivou *et al.*, 2021). The research question (RQ) this study intends to address following an evidence-based approach is:

*RQ: Which are the most important data points of an IS platform (concerning users, materials, energy, water, flows, etc.) and how can they be used for the promotion of IS?*

The phrase ‘IS platform’ was defined as one or a combination of multiple information systems facilitating IS, as no explicit definition was found. The term ‘data points’ was used in a broader sense and understood as all the information that determines the identification and development of symbiotic relationships, especially those that are created using digital tools facilitating IS.

**Figure 1.**
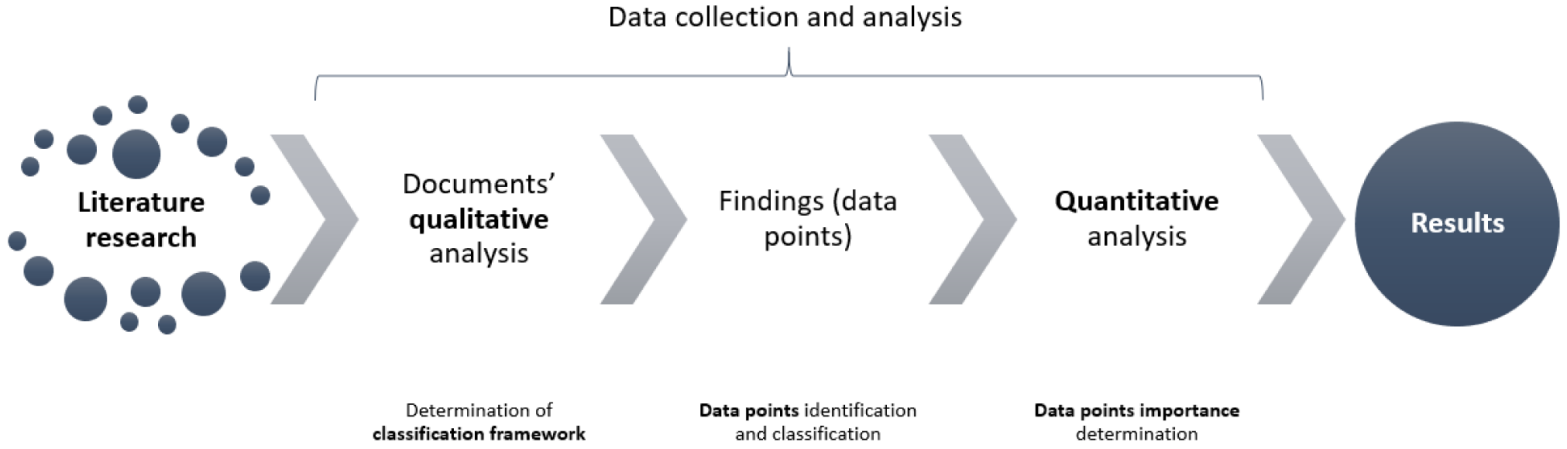
Research framework.

In order to extract the data of interest from the literature in a structured and systematic way, we propose the following research framework ( Figure 1). First, a literature search was conducted, which determined the final manuscripts that were included in the review. Afterwards, data collection and analysis, which was established to extract and organize the results following a qualitative and quantitative analytical approach, was carried out.

### Literature search

In this systematic literature review, the search design was based on a knowledge-based search of keywords, in the form of a query. The fundamental keywords reflecting the concept of the RQ were “industrial symbiosis”, “platform” and “data”; however, since these terms are too general, a structured query with the inclusion of more targeted keywords was designed, resulting in following final query: (“Industrial Symbiosis”) AND (“online platform” OR “digital platform” OR “web platform” OR marketplace OR “ICT tool”). The same query was used for all information sources, scientific databases and grey literature sources. The last day of search for both academic and grey literature was the 9 ^th^ of March 2021. The selected bibliographic databases for academic literature were ScienceDirect, Scopus, SpringerLink, Wiley Online Library, AISel and IEEE (via Google Scholar). The exclusion criteria regarding scientific literature were: i) not written in English language, ii) non peer-reviewed documents, iii) publication date before 2016 and iv) type of document being a book chapter, encyclopedia, editorial, conference abstract, discussion or mini review. SpringerLink conference papers are sometimes a book chapter as well; however, they were included as conference papers. In addition, only fully accessible papers were included from SpringerLink. Moreover, for IEEE, the advanced search engine of Google scholar was used, utilizing the same query with a publication date limit (2016–2021) and with a filter concerning the publication source, IEEE, selected. Grey literature research was based on the customized Google search technique proposed by Godin *et al.* (2015). The same query as for scientific databases was used with a dedicated time frame (2016–2021) and the first 50 results were studied. The following forms of grey literature were excluded: newspaper articles, editorials, book chapters, forums, dissemination papers, business advertisements, student theses, presentations, webinars and interviews, etc. The inclusion criteria for both academic and grey literature were related to the scientific content of each manuscript, namely: i) the document focused on the identification of synergies utilizing ICT tools and techniques, ii) data requirements or platform related information were discussed or iii) the document discussed the impact of a digital tool in promoting IS.

The procedure followed to finalize the papers to be included was configured according to the PRISMA guidelines. The first step was the identification of the relevant scientific publications and documents, both academic and grey literature through the databases and the customized Google search, utilizing the same query. The next step was the removal of duplicates, during which the documents that were identified in more than one source were determined. Afterwards, the first screening of the scientific publications was conducted, during which titles, abstracts and keywords were studied, to remove false positive results derived from the automated search process. Grey literature was not screened during this process, because an abstract was not available in every document. This process was conducted by two independent reviewers, in order to eliminate risk of bias, and automation tools were not employed. Next, the remaining documents considered as relevant from both researchers were fully text read by one of them, reporting to the supervising researcher. The documents that are eventually included in this systematic literature review were selected according to their compliance with the predefined inclusion criteria, as described in the ‘Methods’ section.

### Data collection and analysis

Initially, a qualitative analysis of the included scientific publications and documents was conducted. A classification framework was established in order to obtain and arrange the findings in a systematic and methodological way, according to the sought perspective. The notion behind this classification was to group the papers according to their content, in order to facilitate the data collection and obtain a coherent conclusion.

**Figure 2.**
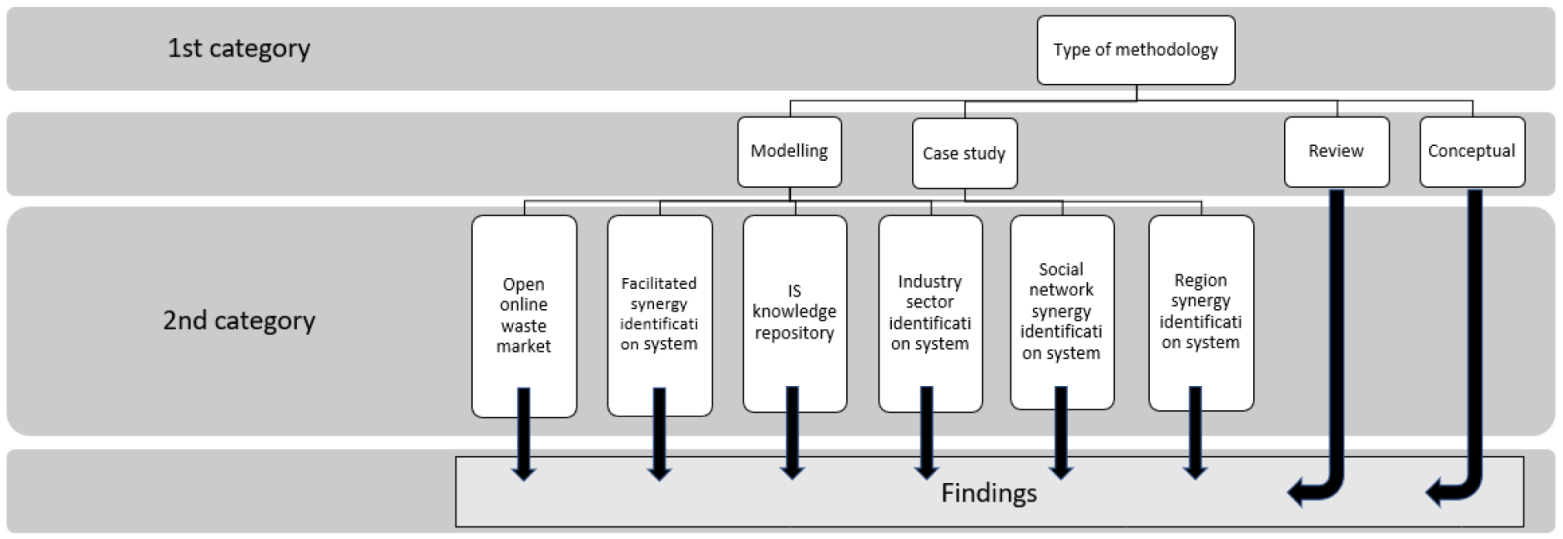
Data collection and findings' extraction methodology.

Each record included in the final study was classified in two different categories by the researcher who performed the full text reading of the documents. The results were reported and discussed with supervising researcher. The first category pertains to the methodology applied in each document, following the categorization methodology proposed by Turken & Geda (2020). The scientific publications and documents were classified according to the type of methodology as “modelling”, “review”, “case study” and “conceptual”. Some documents were assigned to more than one type. The importance of this classification is that papers that fell into the categories “case study” and “modelling”, were further classified according to their content (second category), in order to collect the data. “Review” and “conceptual” papers provide more versatile information and were utilized in a more comprehensive way, validating the results obtained by “case study” and “modelling” papers, as illustrated in Figure 2. 

The second classification category pertains to the content of each paper. The content classification we proposed was based on the type of information system facilitating IS introduced by van Capelleveen *et al.* (2018a), namely open online waste market, facilitated synergy identification system, IS knowledge repository, industrial sector synergy identification system, social network synergy identification systems and region identification system. Each scientific publication and document that belonged to the type of methodology “case study” and “modelling”, was further classified according to the type of information system it described. Some papers were classified into more than one type. The rationale behind the selection of this particular classification framework for classification of the documents from the second category is the intended outcome of the study, namely to collect data and present a literature-based synthesis of the results according to the types of information systems facilitating IS.

That was followed by the identification and classification of data points. Following the classification of the records to the first and the second category, documents from the second category were utilized to qualitatively classify the data points, by identifying patterns to reveal similarities and differences between the types of information systems. Only documents classified in the second category were utilized for this. Initially, we attempted to reveal the application conditions of these systems and an association between the type of information system and the following variables was examined: i) the type of IS network approach (self-organized, top-down or facilitated approach), ii) the size of the network (local, regional, national, or international) and iii) the type of exchange (materials, water or energy).

Furthermore, the method implemented to deduce the results was to associate the type of data points discussed in each document with the type of information system applied. A template was developed to allow qualitative identification, extraction and documentation of data (relevant data points) according to the type of information system facilitating IS. This approach facilitated the identification of data points and assisted the quantitative analysis that followed. Papers classified as “review” and “conceptual” papers from the first classification category were used to confirm data points identified. Since one reviewer curated data without the usage of automation tools or software, consistency was ensured, even though risk of subjectivity is not eliminated.

The quantitative analysis that followed, intended to determine the importance of each type of data point identified. Computational, experimental or any other type of quantitative data was not involved in this literature-based study. The quantification method described in this step pertains to the assessment of data points’ importance. The approach implemented was to detect the number of included studies in the literature review that mentioned it, regardless of the frequency the data point was mentioned in each document. Quantification of data points’ importance was conducted through all documents included in the review. Therefore, number of the data points’ citations was utilized as an indicator of their significance.

Meta-analysis and statistical analysis of the results was not possible, because computational or experimental data were not involved in the included studies. Also, preparation of data, such as handling of missing summary statistics or data conversions was not required and therefore no sensitivity analysis was conducted to assess robustness of the synthesized results, since no actual quantitative data were used. Risk of bias in the results of included studies was not assessed, since only peer-reviewed documents were included. Moreover, there was no direct limitation in terms of geographical scope, but exclusion on the basis of language (the text had to be available in English) provides an element of bias. This risk was addressed by query searching in multiple scientific databases covering several international publishers of multidisciplinary fields and by grey literature retrieval. A risk of reporting bias exists due to one reviewer assessing the eligibility of records to the inclusion criteria. However, the supervision of the second reviewer reduces the risk of personal bias in synthesis of results. Any visual representation of the results was developed by the authors. In the next section results of the literature search on IS platforms and relevant data points are presented.

## Results

**Figure 3.**
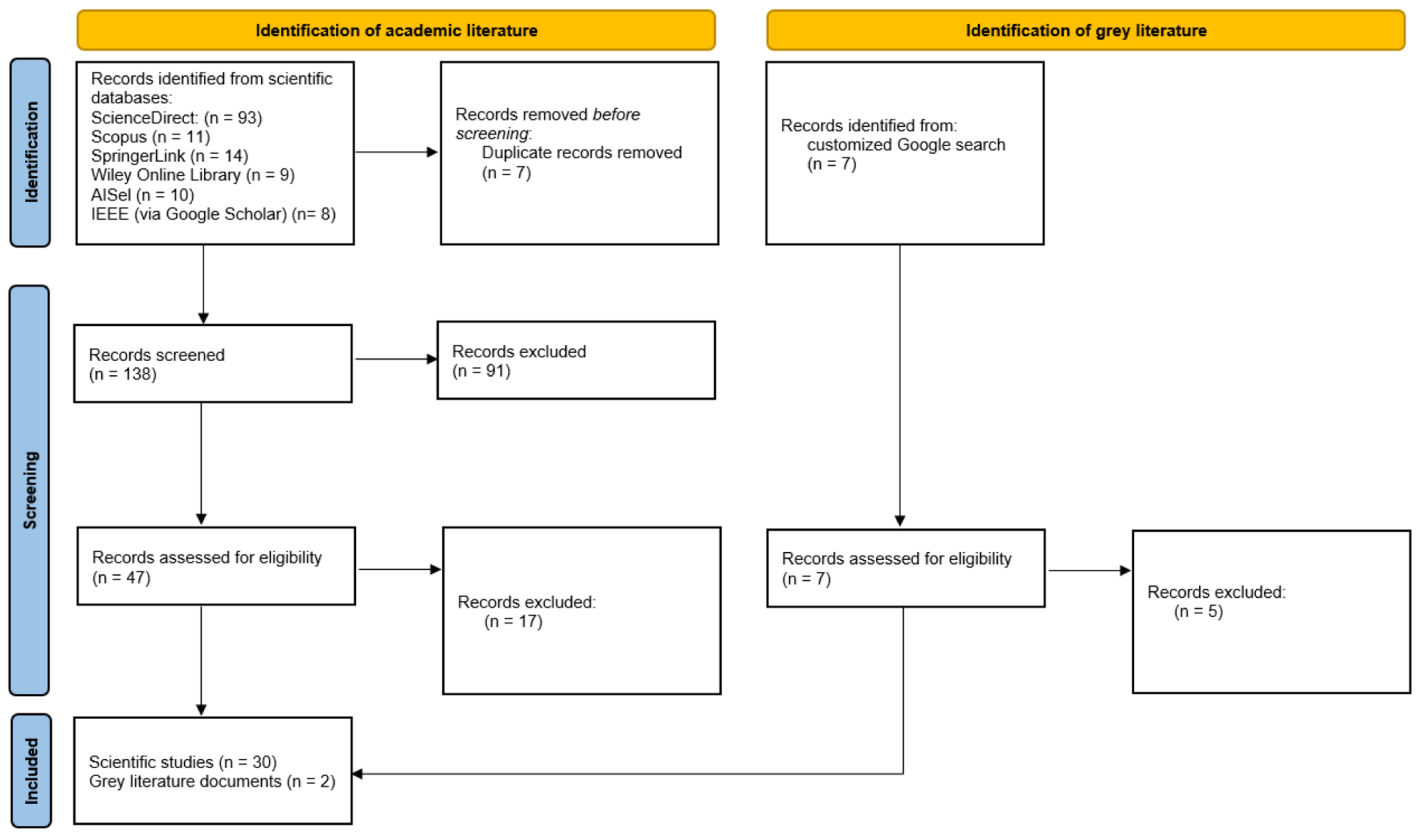
PRISMA flow diagram.

The results presented in this section stem from the documents included in the final research. The total number of records obtained from academic literature was 145 and from grey literature seven. In particular, the number of records identified following the predefined exclusion criteria (see *Methods* section) from ScienceDirect was 93, from Scopus 11, from SpringerLink 14, from Wiley Online Library nine, from AISel 10 and from IEEE (via Google Scholar) eight. After removal of duplicates, the final number of records to be screened was 138. The total number of records excluded during the first screening was 91, resulting in 47 scientific documents to be full text studied and assessed for eligibility according to the presented inclusion criteria. Also, seven grey literature documents remained for full text study as well, therefore resulting in a total number of 54 documents. After full text reading of the 54 documents, 22 were rejected, as their content did not correspond to the predefined inclusion criteria, that is that they did not focus on ICT tools and techniques for the employment of IS and the impact of a digital tool in promotion of IS was not discussed. Finally, the number of scientific publications and documents that were included in this review was identified; a total of 32 records, of which two are grey literature and 30 belong to academic literature. The stages and the number of records at each stage followed are presented in the PRISMA flow diagram in Figure 3.

The findings were analyzed in the context of the proposed classification framework. The categorization of the scientific publications and documents in the two proposed categories is demonstrated. The first categorization was according to the type of methodology the paper employed. As demonstrated in Table 1, each scientific publication and document was classified as “modelling”, “review”, “case study” and “conceptual” based on the details of the methodology applied. The studies that were classified as “case studies” and “modelling” were further analyzed based on their content in the second categorization. The documents that were classified as “review” and “conceptual” provided more broad information, and were not specifically describing a tool that could be classified according to the type of information system applied to facilitate IS. The valuable information provided by these types of documents were used to validate the results obtained from “case study” and “modelling” papers. The only conceptual paper that was classified content-wise was by Ghali *et al.* (2016), because it described a certain type of information system that facilitates IS. Additionally, the only review that was classified content-wise was by Yeo *et al.* (2019b), because it described two types of information systems facilitating IS.

**Table 1.** Scientific publication and document classifications according to type of the methodology.

Type of methodology	Scientific publications and documents
**Case study**	Bakogianni *et al.*, 2019; Fric & Rončević, 2018; Holgado *et al.*, 2018; Holgado *et al.*, 2019; Hsien *et al.*, 2020; John Nurminen Foundation, 2018; King *et al.*, 2020; Kosmol & Leyh, 2019; Kutsikos, 2018; Luciano *et al.*, 2021; Lütje *et al.*, 2019; Lütje *et al.*, 2018; Marconi *et al.*, 2018; Stéphane *et al.*, 2019; van Capelleveen *et al.*, 2021; Yeo *et al.*; 2019a; Zhang *et al.*, 2017
**Conceptual**	Benedict *et al.*, 2018; Font Vivanco *et al.*, 2019; Ghali *et al.*, 2016; Romero *et al.*, 2017
**Modelling**	Fraccascia, 2020; Fraccascia & Yazan, 2018; Fraccascia *et al.*, 2020; Sun *et al.*, 2017; van Capelleveen *et al.*, 2018b; Zhang *et al.*, 2017
**Review**	Halstenberg *et al.*, 2017; Kosmol & Leyh, 2020; Kosmol, 2019; Lawal *et al.*, 2021; Lütje *et al.*, 2019; Lütje *et al.*, 2018; van Capelleveen *et al.*, 2018a; Yeo *et al.*, 2019b

The second categorization of the documents, and the context according to which the findings are further classified and discussed, is based on the types of information systems facilitating IS, as introduced by van Capelleveen *et al.* (2018a). Results regarding the application context of each type of information system result from this research. The type of IS network approach (self-organized, top-down or facilitated approach), the size of the network (local, regional, national, or international) and the type of exchange (materials, water or energy) that the digital tool is designed to facilitate is examined.

Most of the documents that report an open online waste market type of information system, as demonstrated in Table 2, do not specify the IS network approach the tool facilitates. Of those that do, a self-organized network is the most cited approach. However, as Yeo *et al.* (2019b) mention, this type of online platform is also applied in facilitated and top-down IS network approaches ( Yeo *et al.*, 2019b). Although the size of the network is not usually mentioned, regional scale networks are the most common ( Fric & Rončević, 2018; Kutsikos, 2018; van Capelleveen *et al.*, 2018b). Finally, most of the papers consider material exchanges, with energy and water exchange being discussed less in the analyzed literature. In some cases, the information about the type of exchange is missing ( Fraccascia, 2020; Fric & Rončević, 2018; Yeo *et al.*, 2019b).

**Table 2.** Open online waste markets.

Scientific publications and documents	Type of IS network approach	Size of IS network	Type of exchange
**Bakogianni *et al.*, 2019**	Not specified	Not specified	Water, energy, materials
**Fraccascia, 2020**	Self-organized	Not specified	Materials, energy
**Fraccascia & Yazan, 2018**	Self-organized	Not specified	Materials, energy
**Fraccascia *et al.*, 2020**	Self-organized	Not specified	Not specified
**Fric & Rončević, 2018**	Not specified	Regional-national	Not specified
**John Nurminen Foundation, 2018**	Not specified	Not specified	Materials
**Kutsikos, 2018**	Not specified	Regional	Materials (solid)
**Luciano *et al.*, 2021**	Not specified	Not specified	Materials
**Marconi *et al.*, 2018**	Not specified	Regional	Materials
**Sun *et al.*, 2017**	Not specified	Not specified	Materials
**van Capelleveen *et al.*, 2018b**	Facilitated	Regional	Materials
**Yeo *et al.*, 2019b**	Self-organized, facilitated, top-down	Not specified	Not specified

IS, industrial symbiosis.

The scientific publications that portray facilitated synergy identification information systems are demonstrated in Table 3. All the tools reported were utilized in the context of a facilitated or top-down IS network approach. The size of the network they attempt to cover is primarily on a local scale, considering EIPs, but regional scale is mentioned as well. This type of system mainly facilitated materials and energy exchange.

**Table 3.** Facilitated synergy identification systems.

Scientific publications	Type of IS network approach	Size of IS network	Type of exchange
**King *et al.*, 2020**	Facilitated	Regional	Materials
**Kosmol & Leyh, 2019**	Top-down	EIPS	Materials, energy, water
**Lütje *et al.*, 2019**	Top-down/facilitated	EIPS (local)/regional	Materials, energy
**Lütje *et al.*, 2018**	Top-down/facilitated	EIPS (local)/regional	Materials, energy
**Yeo *et al.*, 2019b**	Self-organized, facilitated, top-down	Not specified	Not specified

EIPS, eco-industrial parks; IS, industrial symbiosis.

The papers that describe an IS knowledge repository are shown in Table 4. These systems are not applied within a specified type of IS network approach. The utilization of such systems is envisaged to affect a broad network, therefore even international scale is considered ( Stéphane *et al.*, 2019). These systems include the consideration of materials, water and energy exchange. The two case studies that describe industrial sector synergy identification systems ( Stéphane *et al.*, 2019; van Capelleveen *et al.*, 2021) also describe IS knowledge repositories; therefore, they are further discussed in the context of IS knowledge repositories, unless a difference in the results regarding the type of information system and the type of data points identified occurs.

**Table 4.** IS knowledge repository and industry identification type of Information System.

Scientific publications	Type of IS network approach	Size of IS network	Type of exchange
**Holgado *et al.*, 2019**	Self-organized	Local/regional	Materials, energy, water
**Hsien *et al.*, 2020**	Not specified	Not specified	Water
**Stéphane *et al.*, 2019**	Not specified	International	Materials, energy, water
**van Capelleveen *et al.*, 2021**	Not specified	Not specified	Materials
**Yeo *et al.*, 2019a**	Not specified	Not specified	Not specified

IS, industrial symbiosis.

The only paper that reported a social network synergy identification system was Ghali *et al.* (2016), which was applied to all types of IS network approaches. The size of the network that the tool facilitates is not specified, but it is mentioned that only materials exchange is supported. Additionally, the only tool that was classified as an IS region identification system was the SWAN platform, because of a feature it encompasses that allows regional authorities to participate and manage the synergistic network. However, the SWAN platform will be discussed in the context of open online waste markets, as it predominantly assists the automated matching of stakeholders ( Kutsikos, 2018).

Three papers were found ( Holgado *et al.*, 2018; Lawal *et al.*, 2021; Zhang *et al.*, 2017) that did not belong to any of the information system types. The type of tool or technique described in these papers focused on process integration and process efficiency, utilizing ICT techniques. However, they provide important information on energy exchange and the physical design of an IS network. Those papers are discussed separately, at the end of the section.

### Data point classification

**Figure 4.**
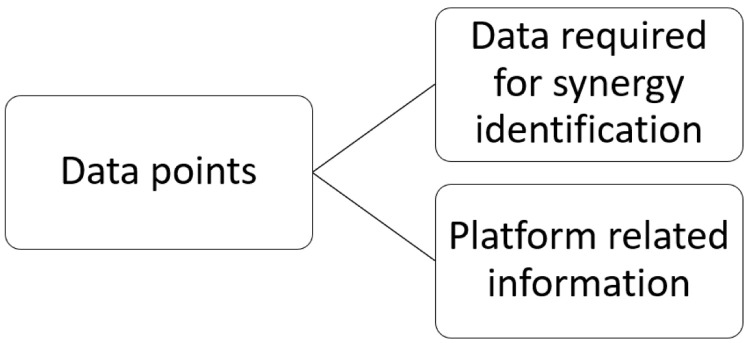
Data points classification.

The systematic extraction of information through the qualitative-quantitative analysis of the selected literature has revealed that within the IS platforms two types of critical data points are encountered, as illustrated in Figure 4. Both types influence the identification and promotion of IS opportunities and are discussed separately due to different features they encompass. The first type concerns the data required for identifying and matching potential synergistic opportunities. The second type is related to crucial information regarding the platform that affect its functionality, and consequently the success, of an IS initiative assisted by a digital tool.

**Figure 5.**
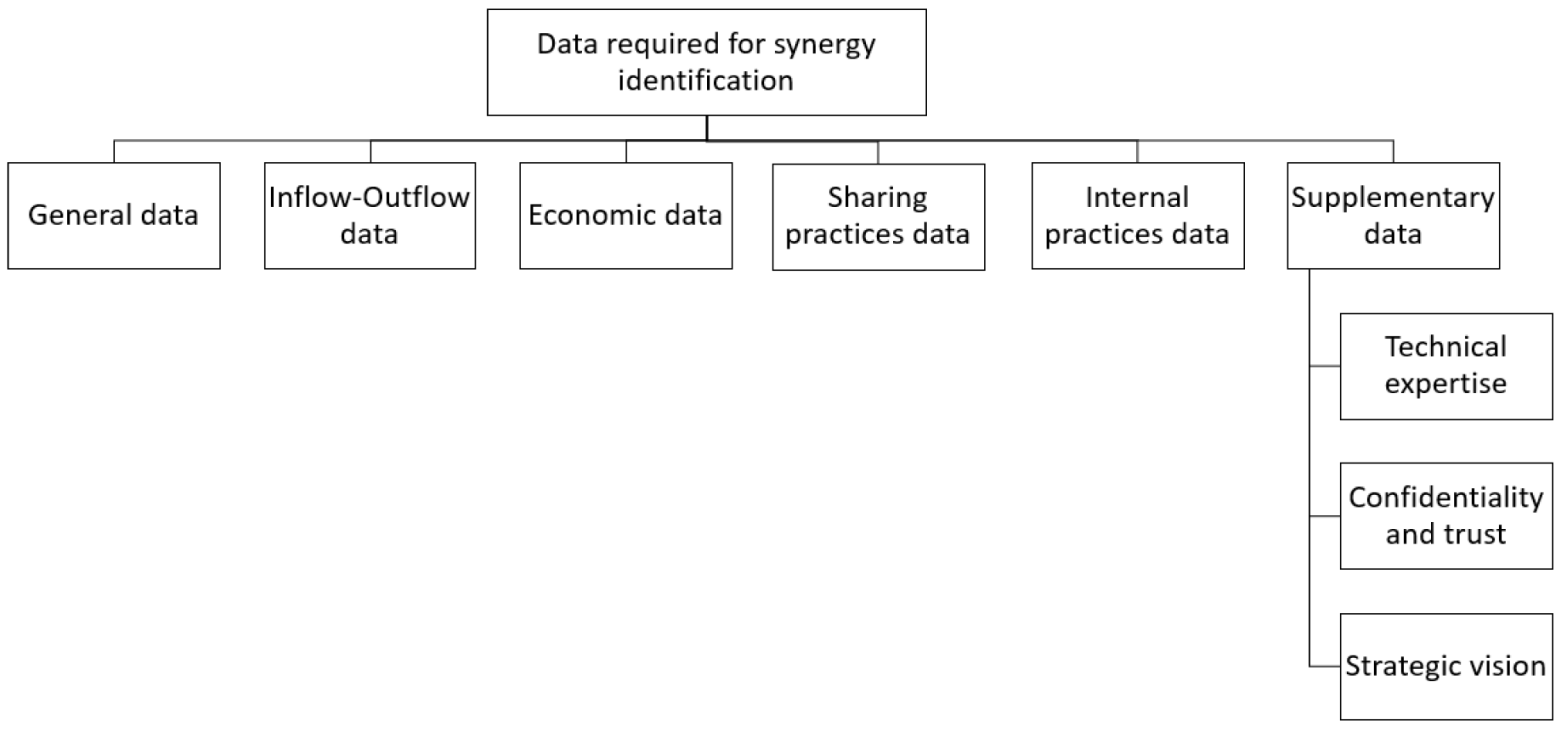
Subcategories of data required for synergy identification.

***Data required for synergy identification.*** Regardless of the type of information system utilized for the realization of IS, certain information is necessary for the accurate detection and overall feasibility estimation of potential matchmaking endeavors. Six different categories were distinguished and those are general data, inflow-outflow data, economic data, sharing practices data, internal practices data, and supplementary data, as demonstrated in Figure 5.

*General data*

Some of the most fundamental, and usually non-sensitive information, is the company’s general data. Especially in the context of open online waste markets, some of the most important data requested by the user are the name and the contact person responsible for the management of the symbiotic relationship ( Fric & Rončević, 2018; Kutsikos, 2018), the exact location of the company (longitude and latitude) ( Bakogianni *et al.*, 2019; Fraccascia, 2020; Fraccascia *et al.*, 2020; Fraccascia & Yazan, 2018; Fric & Rončević, 2018; John Nurminen Foundation, 2018; Kutsikos, 2018; Luciano *et al.*, 2021; Marconi *et al.*, 2018; Sun *et al.*, 2017; van Capelleveen *et al.*, 2018b; Yeo *et al.*, 2019b), the company’s role in the supply chain ( Bakogianni *et al.*, 2019), the industrial sector type according to the proposed taxonomy ( Bakogianni *et al.*, 2019; Fraccascia, 2020; Fraccascia *et al.*, 2020; Fraccascia & Yazan, 2018; Kutsikos, 2018) and the size of the company (number of employees) ( Kutsikos, 2018). Out of the general data listed, exact location is considered the most important, followed by the type of industrial sector the company appertains to and the company’s information (name and contact person). The company’s role in the supply chain is also a key aspect. The same data are also requested in facilitated synergy identification systems, as well as the number of years the company has been operating ( Kosmol & Leyh, 2019). In IS knowledge repositories and industry sector synergy identification systems, the type of general data the user is required to provide are only the company’s role in the supply chain as a receiver or a supplier and the industrial sector type ( Stéphane *et al.*, 2019; van Capelleveen *et al.*, 2021), since the user in this case explores technically feasible synergistic opportunities, not actual symbiotic partners; therefore, more specific data is not requested. Finally, in a social network synergy identification system the company’s name is part of its profile, and the location and industrial sector type are requested ( Ghali *et al.*, 2016).

In all of the documents describing tools facilitating IS analyzed, the users are expected to follow a common terminology regarding the industrial sector type, in order to avoid inconsistency and incertitude. It is particularly important to be compliant with a specified taxonomy, which is frequently combined with a designated classification code. The proposed classification is indicated by the platform in the form of a drop-down list or a textual addition following a certain codification system proposed by the platform provider. The most commonly used classification code in Europe is the Classification of Economic Activities in the European Union (NACE). Other national and international classification codes deriving from international statistical economic activities are also used ( Kutsikos, 2018; van Capelleveen *et al.*, 2021).

*Inflow-outflow data*

To identify possible synergies, it is imperative to gain an insight into the company’s existing production patterns. In open online waste market systems, the user is requested to provide needs and requirement specifications of the demanded and/or supplied resource streams. Initially, the user identifies the supplied or demanded waste stream, for which specific information is requested. The stream could be defined as material, water, or energy flow. For materials, the physical state (solid, liquid, gas) is also requested ( Bakogianni *et al.*, 2019; Fraccascia, 2020; Fraccascia *et al.*, 2020; Fraccascia & Yazan, 2018; John Nurminen Foundation, 2018; Kutsikos, 2018; Lütje *et al.*, 2018; Lütje *et al.*, 2019; Marconi *et al.*, 2018; Sun *et al.*, 2017; van Capelleveen *et al.*, 2018b; Yeo *et al.*, 2019b).

The resource stream’s type and classification are crucial to describe the resource stream and therefore the user is frequently requested to characterize the stream ( Bakogianni *et al.*, 2019; Fraccascia, 2020; Fraccascia & Yazan, 2018; Fric & Rončević, 2018; John Nurminen Foundation, 2018; Kutsikos, 2018; Lütje *et al.*, 2019; Marconi *et al.*, 2018; van Capelleveen *et al.*, 2018b). It is suggested that material and wastewater characterization follow a certain classification code. The most commonly used code in Europe is the European Waste Catalogue (EWC) ( Kutsikos, 2018; van Capelleveen *et al.*, 2018a; van Capelleveen *et al.*, 2021). According to this classification, each waste type is characterized by a six-digit code that indirectly refers to the type of industry, the type of material and type of processes involved in the production of a certain waste stream ( Kutsikos, 2018). Moreover, quantity and quality are important information requested by the stakeholders because they determine the practicality of the potential IS collaborations. This type of information might be considered sensitive by some users, but it is necessary for effective matchmaking ( Kosmol & Leyh, 2019).

The quantity of the flows, both supplied and demanded, is one of the most significant factors influencing a collaboration; therefore, the user is asked to specify the amount (weight or volume) of a stream and to define its physical unit (i.e., kg, m ^3^, etc.). Also, it is suggested that a temporal unit (i.e., per day, per month, etc.) is requested, in order to determine the average amount produced. Apart from quantity, and considered to be less important, is the availability of resources during a specific period. Therefore, along with the amount of production of a certain waste stream, occasionally it is necessary to define its supply pattern, to ascertain the seasonal availability of the stream ( Bakogianni *et al.*, 2019; Fraccascia, 2020; Fraccascia *et al.*, 2020; Fraccascia & Yazan, 2018; Fric & Rončević, 2018; John Nurminen Foundation, 2018; Kosmol, 2019; Kosmol & Leyh, 2019; Kutsikos, 2018; Lütje *et al.*, 2018; Lütje *et al.*, 2019; Marconi *et al.*, 2018; Sun *et al.*, 2017; van Capelleveen *et al.*, 2018b; Yeo *et al.*, 2019b).

The waste’s quality, properties and characteristics is a group of information that might be considered confidential, because it might reveal possible product innovations. However, the technical and regulatory feasibility of the synergistic action often relies on that sensitive data and agreements might be hindered by the lack of such data. The quality compatibility between supplied and demanded resources determines the effectiveness of matchmaking. The most cited quality characterization parameters are composition, ingredients, physical and chemical properties, and potential hazardousness. However, instead of exact values, a range is requested, since wastes and by-products, on the contrary to industrial main products, do not have standard quality, i.e., diversity in properties and chemical composition often emerges. These fluctuations depend on seasonal variations, changes in raw materials and process conditions and the source of the waste stream. Therefore, the resources’ source is a factor that could indirectly affect the intrinsic characteristics of a stream and potential matchmaking as well. Users are required to specify the source of the stream; for example, the specific process that takes place, the conditions of that process, possible pre-treatment and whether it is used as a utility stream (cooling water, steam, process heat etc.) ( Bakogianni *et al.*, 2019; John Nurminen Foundation, 2018; Luciano *et al.*, 2021; Lütje *et al.*, 2018; Lütje *et al.*, 2019). Generally, in open online waste markets, detailed information about the supplied stream and its quality is considered to motivate buyers and lead to prosperous implementation of IS ( van Capelleveen *et al.*, 2018a). 

The same type of information regarding inflows and outflows as in open online waste markets is required for matchmaking in facilitated synergy identification systems. The data collection procedure, however, depends on the facilitation program and its strategy. Such programs are driven by governmental, research or private entities and aim for the organization and coordination of an IS network through an intermediary. The level of guidance, the level of information dissemination and the method that will be utilized in each case depends on the intermediary and the program. For example, in some cases the data is gathered through surveys answered by the stakeholders ( King *et al.*, 2020; Kosmol & Leyh, 2019), through workshops, or it is requested of the users via the platform in the same way as in open online waste markets. An important feature of these systems is that a more comprehensive approach in comparison to open online waste markets is utilized, encompassing all process input and output flows and not only the particular streams the user wishes to indicate ( Yeo *et al.*, 2019b).

IS knowledge repositories and industry sector synergy identification systems are utilized by the end user differently. The user is not requested to provide detailed information of the stream or specific industrial operating data that might appertain to confidentiality. However, the user is expected to identify and select the type of waste according to the classification system that is applied in the platform. In this case, theoretically feasible synergies are proposed based on generic data about the sector and the type of waste, regarding information about the included processes, the average quality in terms of elemental composition and physicochemical properties of input and output resources. However, for accurate opportunity identification, the user is expected to be aware of the company’s produced wastes and by-products and to be able to correctly identify the technical characteristics of the demanded or supplied resource. This way, the valorization opportunity yielded by the system is actually a viable solution, and further investigation for neighboring potential collaborators is carried out by the user, not by the platform ( Holgado *et al.*, 2019; Hsien *et al.*, 2020; Stéphane *et al.*, 2019; van Capelleveen *et al.*, 2021). In the case of a social network synergy identification system, the user identifies the supplied or demanded resources, availability, quantity and quality in terms of potential contamination in order to be automatically matched with a potential partner by the platform ( Ghali *et al.*, 2016).

*Economic data*

Economic data is usually considered sensitive information, and confidentiality issues arise. Nevertheless, it has been shown that sharing additional cost-related information generates IS synergies with greater economic and environmental performance ( Fraccascia & Yazan, 2018).

Considering that open online waste markets enable transactions between companies, the economic viability of the proposed collaborations is an important aspect affecting the promotion of IS and the success of the platform. Therefore, the most important economic data identified is the selling or buying price of the supplied or demanded stream, respectively ( Fraccascia, 2020; Fraccascia *et al.*, 2020; Fraccascia & Yazan, 2018; Luciano *et al.*, 2021; Marconi *et al.*, 2018; Sun *et al.*, 2017; Yeo *et al.*, 2019b). Apart from that, other user’s input economic data concern all the costs before IS implementation and is related to, for example, purchasing primary input materials or treating and discharging waste streams. The most important of those, which are to be defined by the user, are the current waste disposal costs, the waste treatment costs and the cost of buying primary input materials (for the processes for which input is to be replaced) ( Fraccascia, 2020; Fraccascia *et al.*, 2020; Fraccascia & Yazan, 2018; Kutsikos, 2018; Marconi *et al.*, 2018; Sun *et al.*, 2017; Yeo *et al.*, 2019b). At the stage of synergy identification, more costs arise from the formation of a synergistic relationship such as waste transportation and treatment costs. After a match between two companies is identified, it is important to determine a strategy for sharing the additional costs. This could be negotiated between the supplier and receiver after synergy identification or renegotiated in the case of an already established IS relationship. Willingness to share formation costs and the fraction of cost-sharing by each user is a factor uncovering the viability of the synergy formation ( Fraccascia, 2020; Fraccascia *et al.*, 2020; Fraccascia & Yazan, 2018; Sun *et al.*, 2017).

In facilitated synergy identification systems, economic data related to input-output streams are also required. In some cases, the same type of economic data is requested by the user as in open online waste markets ( King *et al.*, 2020; Kosmol & Leyh, 2019). However, in other cases, a more systematic cost analysis of the participating companies takes place, utilizing advanced tools and methods ( Lütje *et al.*, 2018; Lütje *et al.*, 2019).

In IS knowledge repositories, sensitive economic data are not required by the user. However, a cost estimation takes places based on generic information so that the suggested opportunities are financially evaluated ( Hsien *et al.*, 2020). In social network synergy identification systems, the user determines the selling or buying price of the supplied or demanded resources ( Ghali *et al.*, 2016).

*Sharing practices data*

Even though IS is associated with excess material, water and energy exchange, it also concerns inter-firm infrastructure, utilities and service sharing. This type of synergy incorporates more than a mere exchange of products, it includes potential sharing of facilities, i.e., physical spaces (storage, warehouses etc.), and services, i.e., logistics (transportation vehicles), cleaning, security, disposal/treatment of wastes, staff training, personnel skills, experience, and knowledge, etc. In addition, joint use of technical infrastructure such as machinery, pipelines, pumps, ICT equipment etc., and utilities such as heat, power, steam flows, water etc. is considered. Sharing practices data are rarely considered in open online waste markets, and never considered in IS knowledge repositories, industry sector synergy identification systems, or social network synergy identification systems. However, in facilitated synergy identification systems and, especially at a local scale, joint usage of infrastructure and utilities are taken into account. Users of the platform are requested to identify the type of infrastructure, service, or utility they are interested in sharing with nearby companies, usually in an EIP or within a region, and the period they are willing to share it. Additionally, users are expected to report any existing shared infrastructure to the platform ( Lütje *et al.*, 2018; Lütje *et al.*, 2019; Romero *et al.*, 2017).

*Internal practices data*

A significant aspect influencing a company’s management involves the internal practices and methods for mapping, monitoring, gathering information, evaluating, and documenting of industrial processes and applied supply chain model. Sharing this type of information is considered a part of a more sophisticated, detailed and coordinated form and requires a high level of trust from the stakeholders in the platform provider. Therefore, internal practices data is predominantly regarded in facilitated and top-down IS network approaches and in retrofitting of an industrial park into an EIP ( Kosmol & Leyh, 2019; Lütje *et al.*, 2018; Lütje *et al.*, 2019; Yeo *et al.*, 2019b).

Several certifications can provide an insight into the company’s standard procedures regarding manufacturing practices, process monitoring, environmental and supply chain management, and documentation. Hence, any energy/environmental ISO certifications or supply chain management certifications can be provided by the user to the platform. This type of information can reveal the company’s practices related to water, energy, material consumption and waste generation and management, which indicates the readiness and compatibility of possibly future collaborating industries, to effectively implement IS. It also discloses information about internal tools, methods and technologies utilized in a company. These tools, methods and technologies are often standardized according to the management systems’ certifications; consequently, they are a corollary of the company’s certifications. These methods are important because they provide data regarding different aspects of the product’s lifecycle that could be utilized by an IS platform, if available ( Halstenberg *et al.*, 2017; Kosmol & Leyh, 2019; Lütje *et al.*, 2018). Some frequently applied methods for material, water and energy input and output flow tracking include Material Flow Analysis (MFA), Material Flow Cost Accounting (MFCA) for assessing their associated value and Life Cycle Analysis (LCA) for evaluating the environmental impact. Some of the organizational data systems utilized by a company for management of the production processes, the supply chain, and other services include environmental management system (EMS), enterprise resource planning (ERP) systems, and supply chain management (SCM) systems etc. ( Halstenberg *et al.*, 2017; Lütje *et al.*, 2018; Lütje *et al.*, 2019). The boundaries for such tools and techniques to be applied are not restricted; they are carried out at entity-level or network-level for a certain synergy (match) or for a total site (EIP) ( Lütje *et al.*, 2018; Lütje *et al.*, 2019).

*Supplementary data*

The data described in the above sections are easily captured, coded, quantified, and communicated as actual data by the user. However, more complex, qualitative information about technical aspects, as well as the companies’ incentives, relational dynamics and overall institutional capacity is also necessary. In this context, supplementary data pertain to occasions where the stakeholder’s subjective point of view is considered, which in some cases is equally important. In order to extract such information, surveys, questionnaires or textual descriptions by the user are utilized in digital tools. Such approaches are used in open online waste markets, facilitated synergy identification systems and social network synergy identification systems, where users seek suitable collaborators. In knowledge repositories such data are not considered ( Ghali *et al.*, 2016; Kosmol, 2019; Kosmol & Leyh, 2019; Yeo *et al.*, 2019b).

### Technical expertise

Technical knowledge, expertise and experience of a company is generally considered complex and difficult to communicate; however, it is crucial for operational achievement of IS, for determining valorization pathways and for removing technical feasibility barriers. The technological expertise might concern several aspects of the company and its products including processes, equipment, experienced and specialized personnel as well as waste, by-product or additional product specifications. Therefore, some platforms envisage using such knowledge to match optimal synergistic partners, where technology is provided as a part of the supply chain and collaborators are also identified according to their available expertise ( Bakogianni *et al.*, 2019; Kosmol, 2019; Marconi *et al.*, 2018).

In some cases, codification of waste/by-product is not adequate to describe a product and its features; therefore, the user is requested to provide extra information regarding the supplied stream or product. Such addition of textual information is often encountered in the discrete parts and product manufacturing industry, where the output stream is non-continuous and the products are items that differ from each other. For example, parts of different size, geometrical shape, color, coating, thickness, etc., might need to be identified and grouped by the provider, and afterwards reported to the platform. In process industry, the waste classification system might enclose overlappings in certain taxonomies; therefore, a short word description by the user might be necessary. Moreover, it gives the user an opportunity to offer other possibly valuable information concerning technical specifications of these by-products, such as pre-treatment, pre-processing, utilization requirements in further processes etc. as well as description of any existing technical and environmental standards or certifications of the material ( Halstenberg *et al.*, 2017; John Nurminen Foundation, 2018; Kutsikos, 2018; Luciano *et al.*, 2021; van Capelleveen *et al.*, 2018b; Yeo *et al.*, 2019b).

Moreover, information about the company’s existing waste management methods and available technologies, as well as equipment, are requested in some cases. The identification and description of the user’s expertise in manufacturing and service technologies is requested, in order to match suitable stakeholders and suggest indirect matches that otherwise would be infeasible or undetected ( Bakogianni *et al.*, 2019; Kutsikos, 2018; Marconi *et al.*, 2018). For example, according to Kutsikos (2018), the S.W.A.N. platform includes a data point with regards to a company’s current management method of waste streams and current supply method of input streams, where a user has the option to choose from an existing list or describe the method via textual addition. The user is also requested to state whether waste management is carried out by an external stakeholder and if the necessary infrastructure or storage facilities are available ( Kutsikos, 2018). Moreover, as described in Marconi *et al.* (2018), the user is requested to state the available technological equipment and expertise, in order to be used for direct or indirect matches with other stakeholders ( Marconi *et al.*, 2018).

In the case of social network synergy identification systems, sharing of information, knowledge and experiences is an approach used to demonstrate potential technical expertise of a member and to promote the company and its accomplishments. It also provides a communication channel for users to collectively develop further knowledge and to assist each other by sharing related IS experiences, a process that helps overcome potential difficulties that occur during IS practices ( Ghali *et al.*, 2016).

### Confidentiality and trust

When identifying and assessing the potential symbiotic relationships, it is particularly important to rely on as much information as possible. Sensitivity of data is a profoundly subjective issue, and each user is requested to classify the disclosed data in regard to confidentiality. In addition, it is necessary to define the extent to which the data will be available and accessible. In order to assure a competitive advantage, companies may seek to prevent other companies from having access to confidential information, and only the platform provider may have direct insight to selected data ( Benedict *et al.*, 2018; Fraccascia & Yazan, 2018; Ghali *et al.*, 2016; Kosmol, 2019; Kosmol & Leyh, 2019; Lütje *et al.*, 2018; Lütje *et al.*, 2019).

The willingness of stakeholders to cooperate and share information is one of the most significant factors promoting the implementation of IS. Willingness to share information is related to trust among stakeholders, which is often associated with social relationships. A list with existing symbiotic relationships and any involvement with IS initiatives is, in some cases, requested by the users of the platform. In addition, a list of commercial connections is required in order to identify potential existing linkages ( King *et al.*, 2020; Kosmol, 2019; Kutsikos, 2018). Moreover, King *et al.* (2020), requested a list of personal relationships with actors from other companies participating in the IS network. Through this research, it has been proven that pre-existing personal relationships boost the formation of business relationships within an IS network. Additionally, high trust in a partner coming from previous cooperative interaction can surpass even the economic incentive, the so-called ‘path dependence theory’, proving that social aspects are just as important in business transactions as financial issues ( Fraccascia, 2020; Fraccascia *et al.*, 2020; Fraccascia & Yazan, 2018).

### Strategic vision

Managerial aspects of IS participants affects the strategy they pursue and the extent of IS adoption. The understanding of the possible involved entities’ incentives and readiness to foster symbiotic endeavors is an important element, which in many cases is being neglected. Successful implementation of IS and strengthening of symbiotic relationships to create a resilient network is significantly influenced, among other factors, by the long-term vision and plans of each company. The notion is that synergies will be established between members that share the same perspective and the most compatible actors will be identified by the platform and linked ( Kosmol, 2019; Kosmol & Leyh, 2019; Lütje *et al.*, 2019; Marconi *et al.*, 2018; Yeo *et al.*, 2019b).

Interests and incentives of companies to engage in sustainable business models are not easily measurable or controllable. Different driving forces for companies to join an IS network exist. The drivers might be economic, environmental, social, or a combination of those. Recommended synergies by the platform should be attractive enough for the user that they consider forming a symbiotic relationship. The most predominant driver for a company is usually the economic benefit; however, it is not unlikely that a company will seek the synergy with the lowest environmental impact, either because it is environmentally conscious or regulatory constraints arise ( Benedict *et al.*, 2018; Fraccascia, 2020; Fraccascia & Yazan, 2018; Ghali *et al.*, 2016; Kutsikos, 2018; Sun *et al.*, 2017).

Presently, there is no feature assessing the compatibility of the potential synergy depending on the companies’ managerial aspects. In some cases, scholars attempt to perform a preliminary investigation of the drivers and barriers of an IS network before the implementation of any IS practices. Specifically, surveys and questionnaires that members answer before using the IS platform have been developed, which aim to estimate the participants’ strategic direction and maturity to contribute to the network. The questions address the stakeholders’ familiarity with IS principles, their objective for participating in IS, their perception of successful implementation of IS, their willingness to invest in time and capital, their self-estimation of readiness and maturity to collaborate and commit to sustainable business model and their view on using IS platforms for networking ( Fric & Rončević, 2018; Kosmol & Leyh, 2019; Kutsikos, 2018; Lütje *et al.*, 2019). The only tool addressing such aspects as a compatibility indicator during the identification of potential symbiotic partners is the digital tool proposed by Marconi *et al.* (2018), which envisages to incorporate a technique that links stakeholders with the same strategic vision. However, what the required data for that compatibility assessment is and how that data is collected is not explained further ( Marconi *et al.*, 2018).

**Figure 6.**
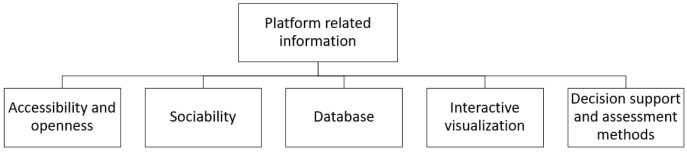
Subcategories of platform related information.

***Platform related information.*** The effective promotion of IS through digital tools is not limited to the identification and matching of the potential partners of an IS network. The features the digital tools encompass and the possibilities they provide to the end-users influence the promotion of long-term symbiotic relationships. Through this research some of the most important information regarding an IS platform is determined. The results are discussed in the context of the types of information systems. Five different categories were distinguished, namely: information related to the accessibility and openness of the platform, the sociability, the database used by the platform, the interactive visualization, and the decision support the platform provides to the users through assessment methods. The classification framework is demonstrated in Figure 6.

*Accessibility and openness*

The number of platform users is extensively associated with platform accessibility and the extent of engagement of companies. The successful promotion of IS through a digital tool is established when the critical mass of users is achieved. It has been proven that as the number of platform users increases, the platform’s effectiveness in matchmaking and assistance in maintaining synergistic relationships increases. The reason for this is that the number of suggestions for potential symbiotic partners is greater, allowing the user to choose from a variety of options. Additionally, the greater the number of relationships for the same waste exchange, the lower the risk and uncertainty of vulnerability to perturbations. A redundant number of symbiotic relationships is recommended in order to create a resilient IS network. Moreover, the number of participating users that act as suppliers and the number of those that act as receivers should be balanced for the supply to meet the demand and for the network to be strengthened ( Fraccascia, 2020; Fraccascia *et al.*, 2020; King *et al.*, 2020).

According to the literature, the number of platform users is related to the type of IS network approach the digital tool is designed to facilitate. The network is limited to a certain number of users when the platform is facilitating EIPs, small industrial areas, or certain research projects, posing a great hurdle to engaging new participants and eventually resulting in an inoperative system. Generally, when a digital tool is designed as a part of a facilitated or a top-down approach, the platform is accessible only to involved entities, being either industrial actors, project partners or members of the EIP. On the contrary, tools promoting self-organized approaches have been designed for commercial usage and are publicly accessible. An openly accessible tool, presumably, has a greater possibility to attract the critical mass of users necessary for the proper operation of the platform ( Benedict *et al.*, 2018; Kosmol & Leyh, 2020; van Capelleveen *et al.*, 2018a). This research revealed that open online waste markets are predominantly openly accessible to businesses to register and become a member ( Bakogianni *et al.*, 2019; Fric & Rončević, 2018; Kutsikos, 2018; Luciano *et al.*, 2021; Marconi *et al.*, 2018; Yeo *et al.*, 2019b), while facilitated synergy identification systems are only accessible to the participants of the program or members of the EIP ( King *et al.*, 2020; Kosmol & Leyh, 2019; Lütje *et al.*, 2018; Lütje *et al.*, 2019).

Nevertheless, the number of platform users is not the only decisive factor in the platform’s viability and success. Another important aspect is the active users’ participation, as it is common for IS platforms to operate with low active user engagement. This is a barrier, especially in open online waste markets, where the IS network is required to perpetually grow and users are required to be relatively active for the platform to remain operational. According to Fraccascia (2020), the establishment of a platform or marketplace alone is inadequate to promote the adoption of IS. The number of users engaging in IS practices through the platform, i.e., usage rate, determines not only the platform’s performance, but also the users’ competitive advantage over non-users. For low platform usage rates, the benefits gained are scant or even nonexistent and non-users do not face competitive disadvantage. However, as the usage rate increases, active users’ favorable position strengthens and non-users deal with incapacity to create and operate IS relationships ( Fraccascia, 2020; Fraccascia *et al.*, 2020; Yeo *et al.*, 2019b).

IS knowledge repositories and industry sector identification systems are designed to provide knowledge and help companies to reveal potential synergies by proposing relevant ideas based on generic data and previous implemented case studies; therefore, they are open to the public ( Holgado *et al.*, 2019; Stéphane *et al.*, 2019; van Capelleveen *et al.*, 2018a; van Capelleveen *et al.*, 2021).

Finally, a social network synergy identification system is considered an open access tool, since any company can create a profile and expand the professional network, create or join IS dedicated groups, share knowledge and information, and collaborate openly with businesses or organizations ( Ghali *et al.*, 2016).

*Sociability*

Social aspects of the developing network are essential for the initiation, progress and sustenance of symbiotic relationships. Consequently, the most relevant types of information systems to sociability are mainly open online waste markets, facilitated synergy identification systems and, undoubtedly, social network synergy identification systems where collaborations with other users are pursued. Many tools lack the element of participants’ social involvement and due to this have been characterized as outdated. Currently, scholars have suggested the integration of a social network module in IS platforms, which assists the formation of relationships among actors and enables their interaction. Additionally, social media and social networks facilitate and promote users’ active participation, since interaction among companies and other users is enabled through this communication channel ( Benedict *et al.*, 2018; Ghali *et al.*, 2016; Kosmol & Leyh, 2020; van Capelleveen *et al.*, 2018a). The most evident example of a tool incorporating social features is the Green Social Network (GSN) introduced by Ghali *et al.* (2016). This system presents an opportunity for companies, in a form of communication channel, to maintain and reinforce pre-existing ties, both business and personal, to discover and develop new cooperative relationships, to share knowledge, information, expertise and ideas among the community and to contribute to reputation building. To some extent, social networks and social media resemble offline IS face-to-face meetings and workshops, with the difference being that the online option does not face as many limitations. For example, offline IS events are confined to a certain number of participants from a particular geographical region bound by their physical presence, whereas an online version is open for all interested parties to join without any location constraints. Some proposed means to achieve creation and expansion of a network in a collective manner are the development of groups, blogs, forums, chat rooms, event calendar etc. on already existing social media (Twitter, Facebook, LinkedIn, etc.), or creation of a social network platform dedicated to IS, that includes some basic required functions. These functions consider the capability of organization and dissemination of information, the capability of contact and discussion in real-time with other members, a search tool for essential information inquest, the disclosure of optative information, and the advertisement of the company and its achievements ( Ghali *et al.*, 2016).

The tool Lütje *et al.* proposed also encompasses an integrated social network platform that enables communication and trust building among stakeholders, knowledge and information sharing, and other community engaging activities ( Lütje *et al.*, 2018; Lütje *et al.*, 2019).

*Database*

One of the main components of a platform is the various databases it incorporates to support the matchmaking of the inflow-outflow of potential collaborators. In open online waste markets, facilitated synergy identification systems and social network synergy identification systems it is particularly important to encompass an external supporting database because it contains background knowledge that determines the suggested synergies. In addition, these databases help to tackle the knowledge gap of technically feasible waste valorization pathways ( Hsien *et al.*, 2020; Kosmol & Leyh, 2020; van Capelleveen *et al.*, 2018b).

The data sources used to populate the external knowledge base predominantly appertain to scientific literature, legislation, reports from national and international organizations, previously implemented IS case studies and patents. The type of data such databases usually include are material flow databases, life cycle inventories, resource substitution data, national pollutant emissions and environmental data sets, best IS practices, available data from IS workshops, IS technologies and technical standards and general information for practitioners ( Bakogianni *et al.*, 2019; Halstenberg *et al.*, 2017; Kutsikos, 2018; Luciano *et al.*, 2021; van Capelleveen *et al.*, 2018a; van Capelleveen *et al.*, 2018b; van Capelleveen *et al.*, 2021; Yeo *et al.*, 2019a). One major challenge concerning literature-based databases is that it is difficult to develop, populate and maintain them since the research output is perpetually growing. However, attention to the expansion of IS databases has been increasing, as their significance in synergies’ identification is evident. Manual collection and handling of textual data is considered impractical and expensive. Therefore, Yeo *et al.* (2019a) proposed a self-learning database that is capable of processing large quantities of text encompassing a Natural Language Processing (NLP) pipeline module. This is a promising method that if embedded into a platform’s database, would possibly reveal more valuable waste valorization pathways and alternative conversions of wastes by utilizing all types of textual information ( van Capelleveen *et al.*, 2018a; Yeo *et al.*, 2019a).

An important aspect of the incorporation of the external database into the IS platform is that a common taxonomy should be followed, especially for explicit knowledge-based recommenders. In that case, for the platform to correlate the information from the external knowledge source, for example a repository of previous case studies, to the data provided by the user, the same dataset and a consistent terminology are essential. Otherwise, a service for taxonomy translation is required, so as to ensure the correlation of the information provided ( van Capelleveen *et al.*, 2018a; van Capelleveen *et al.*, 2018b; Yeo *et al.*, 2019b).

*Interactive visualization of results*

Many scholars promote the representation of suggested synergies and functions of the platform as important features, influencing the promotion of IS through digital tools. Specifically, interactive visualization of results stimulates user engagement in the platform by enabling easier and quicker understanding of information and by guiding them towards specific conclusions. As the usability of the platform increases, the efficiency of the user’s decision-making process intensifies and user satisfaction increases. Such visualization of results is regarded in all types of information systems facilitating IS ( Font Vivanco *et al.*, 2019; Lütje *et al.*, 2018; van Capelleveen *et al.*, 2021).

The most common form of data visualization used in tools facilitating regional or national sized networks are interactive maps, which use information systems such as Geographic Information System (GIS) and other tools that facilitate the comprehension of geographic proximity between two potential partners and calculate the exact distance. Besides this, some systems support the visualization of the economic activity of a region or the contingent transportation costs between two collaborators, to determine the feasibility and profitability of a single relationship or of an entire region ( Bakogianni *et al.*, 2019; Fraccascia, 2020; Luciano *et al.*, 2021; van Capelleveen *et al.*, 2018a; Yeo *et al.*, 2019b).

The representation of material and energy flow exchanges via an interactive approach is also gaining attention, due to increasingly detailed monitoring and control of processes, which enables the discovery of optimization opportunities. The creation of material exchange maps with illustration of different technically feasible paths through color codes, sizes and other visual techniques could support the comprehension of the recommended synergistic relationships. Another data visualization approach proposed to simplify the interpretation of the results is through Sankey Diagrams. Additionally, heat maps are used with the aid of geo-spatial related data and energy related process data ( Font Vivanco *et al.*, 2019; Holgado *et al.*, 2018; Hsien *et al.*, 2020; Lütje *et al.*, 2018; Lütje *et al.*, 2019; van Capelleveen *et al.*, 2021; Yeo *et al.*, 2019b)

Moreover, due to the fact that the number of technically feasible potential collaborations and waste valorization pathways might be extensive, it is essential to assist the user in navigating the platform. Therefore, several functions have been proposed in the literature to ease the user’s experience. Such functions include filtering techniques, allowing the user to select the type of material, region etc. that they seek to gain an insight into and, simultaneously, limiting the information load. Drop-down lists, ‘drag-and-drop’-style tools, checkboxes, search tools and other functions have been proposed to facilitate the user’s exploration. Another function of the platform proposed to unburden the user is the sorting and ranking of the recommended matches according to certain variables. For example, according to Bakogianni *et al.* (2019), the suggested synergies of the platform are sorted in order of driving distance. An approach suggested by Hsien *et al.* (2020), is to allow the user to determine the most significant factor of interest (i.e. cost or environmental impact) and the results are ranked according to that preference from the most to least appealing options ( Bakogianni *et al.*, 2019; Font Vivanco *et al.*, 2019; Hsien *et al.*, 2020; van Capelleveen *et al.*, 2021). Finally, most of the proposed tools are web-based platforms; however, a few scholars have mentioned the likelihood of extension of tools as mobile applications to develop an even more engaging user experience ( Lütje *et al.*, 2018; Lütje *et al.*, 2019; van Capelleveen *et al.*, 2021).

*Decision support and assessment methods*

The matching process between suppliers and consumers depends on the external database of the platform and a set of algorithms, which determine the technically feasible waste conversion pathways. Nevertheless, current digital tools, apart from identifying potential symbiotic opportunities, are expected to provide advanced assistance to the decision makers by demonstrating the impact of the proposed symbiotic links and suggesting the most appealing among various options. Therefore, a tool that evaluates all the possible synergies via different methods and provides recommendations is an important feature of the platform, intended to create incentives and support the users’ decision making ( Benedict *et al.*, 2018; van Capelleveen *et al.*, 2018b; Yeo *et al.*, 2019b).

According to van Capelleveen *et al.* (2018a), facilitated synergy identification systems actively provide decision support to the participants, whereas open online waste markets facilitate the waste transactions in a passive way, not supporting the decision-making of the users ( van Capelleveen *et al.*, 2018a). Nonetheless, current open online waste markets are expected to evolve by incorporating expert systems, such as recommenders, to contribute effectively and dynamically to the expansion of IS networks. According to van Capelleveen *et al.* (2018a), platforms that integrate implicit knowledge-based recommenders, which attempt to detect users’ transactional and behavioral underlying patterns and make matchmaking predictions, perform better at discovering IS opportunities than explicit knowledge-based recommenders. The latter is developed on the concept of utilizing data from external databases. The drawback of this implicit knowledge-based method is that a large number of previous transactions should be available in order for the recommender to be operational ( van Capelleveen *et al.*, 2018b). Moreover, a proposed IS knowledge repository by (Hsien *et al.* 2020), incorporates facilitation of the decision-making by evaluating the operational cost and environmental impact of the recommended waste conversion pathways, confirming that such systems can be integrated in IS knowledge repositories as well ( Hsien *et al.*, 2020).

Typically, a platform’s integrated decision support tool incorporates quantitative performance indicators so that the impact of a potential symbiotic relationship is computed. Following the synergy’s technical feasibility performance evaluation, the most commonly applied predictive performance assessment concerns economic viability and environmental impact. However, social benefits are also important to an IS network, thus the assessment of the social performance as an indicator of a synergistic match or a whole EIP has been proposed as well ( Kosmol, 2019; Yeo *et al.*, 2019b).

With respect to the economic viability evaluation, economic data provided by the user is used in most open online waste markets ( Fraccascia, 2020; Fraccascia *et al.*, 2020; Fraccascia & Yazan, 2018; Kutsikos, 2018; Luciano *et al.*, 2021; Marconi *et al.*, 2018; Sun *et al.*, 2017; Yeo *et al.*, 2019b), whereas in facilitated synergy identification systems a more structured financial assessment of the proposed synergies related to the streams’ value is applied ( Lütje *et al.*, 2018; Lütje *et al.*, 2019). The suggested method for computing the economic benefits takes into consideration the cost savings (reduction in waste discharge costs/purchasing primary input materials costs), the potential transportation and waste treatment costs that arise, and the transaction costs ( Fraccascia, 2020; Fraccascia *et al.*, 2020; Fraccascia & Yazan, 2018; Sun *et al.*, 2017). Other methods of economic analysis include net present value (NPV), internal rate of return (IRR), payback period, incremental investment return (IIR) and return on investment (ROI) ( Yeo *et al.*, 2019b). In the case of the IS knowledge repository Hsien *et al.* (2020) proposed, the cost estimation appertains to the operating cost of the processes included in the recommended waste conversion process pathway ( Hsien *et al.*, 2020).

 With respect to the environmental impact assessment, a variety of methods are employed, mainly considering waste reduction in terms of materials savings and reuse, minimization of energy consumption and related emissions due to production processes and transportation. In any case, environmental impact assessment is based on a comparison between the IS studied scenario and a reference scenario, in which no symbiotic exchanges occur. Direct environmental benefits for the involved entities are predominantly regarded; however, indirect benefits for the entire community can be estimated as well. Direct benefits include materials saving (the amount of waste averted from landfill disposal) or reuse (the amount of original input not used in the industrial processes), whereas indirect benefits include GHG emissions reduction ( Fraccascia, 2020; Fraccascia *et al.*, 2020; Fraccascia & Yazan, 2018; Hsien *et al.*, 2020; Luciano *et al.*, 2021; Sun *et al.*, 2017). Material mapping techniques, such as MFA, are particularly useful to quantify the resource intensity of an IS system and calculate the direct environmental benefits ( Holgado *et al.*, 2018; Lütje *et al.*, 2018). Indirect benefits are more complex to estimate, and more advanced methods are employed, involving several environmental indicators portraying the environmental performance of an IS system. Such methods include LCA and the Carbon Footprint (CFP) methodology. However, since these methods deploy more thorough analysis of the life cycle of a product and the related processes, limitations regarding the level of detail encompassed in relation to each process unit occur. The CFP technique employed by Hsien *et al.* (2020) considers only the environmental impact of the processes involved in waste conversion pathways, whereas more extensive approaches also involve energy consumption, water or ozone depletion. Moreover, another constraint of CFP appertains to the emission factors utilized to quantify the impact of a process, which depend on location information, thus providing divergent results. A limitation regarding LCA is related to the necessity of an external database, which poses extra expenses ( Holgado *et al.*, 2019; Hsien *et al.*, 2020; Lütje *et al.*, 2018; Lütje *et al.*, 2019; Marconi *et al.*, 2018; Yeo *et al.*, 2019b).

Because of the diversity of factors influencing the users’ decision process, a multi-level evaluation has been introduced as a solution. Attempts have been made to utilize the technique of Analytic Hierarchy Approach (AHP), by creating weighted normalized evaluations based on fuzzy logic. The integration of several elements to a single index is a method being considered for the construction of spatial decision support systems facilitating regional IS and for the evaluation of EIPs ( van Capelleveen *et al.*, 2018a; Yeo *et al.*, 2019b).

The demonstration of the potential benefits generated by the synergy is regarded as constructive for the selection of the most suitable partner (or valorization pathway) according to the respective user’s preference. However, other, more complex factors not incorporated in the platform might also affect the user’s final decision on the selection process of synergistic partner. Therefore, the decision support tool is suggested to assist the user up to a certain level, but the final decision relies upon the respective user’s judgment and preference ( Fraccascia & Yazan, 2018).

### Process integration tools and energy exchange

Another type of tool and technique utilized to identify technically feasible synergistic opportunities is Process Integration (PI) tools. These tools utilize mathematical techniques to determine the physical feasibility of the design of an IS network and its optimization according to the objective. PI tools can identify optimal matches depending on the optimization goal and they are particularly useful for either the systematic planning and realization of new EIPs or the transformation of an industrial park to an EIP. PI techniques were initially applied for intra-firm physical network design, optimization and cost reduction. Their application in IS aims at minimizing resource (water, energy or materials) or fuel consumption, reduction of capital, operational, utility costs or GHG emissions, increasing process efficiency and enhancing the implementation of resource exchange. The optimization goal may vary, and several different mathematical computer-aided models have been utilized to achieve network optimization when it comes to water, heat, power, GHG emissions and material interplant integration. Ordinarily PI techniques focus on the optimization of one variable; however, multiple variable optimizations might occur concurrently. Ontologies have been proposed as a promising approach to incorporate PI techniques into ICT tools by defining and relating domain concepts and using sets of semantic statements. This incorporation is envisaged to advance inter-firm exchange of resources to create knowledge-based systems for IS identification, especially for thermal energy exchange ( Lawal *et al.*, 2021; Yeo *et al.*, 2019b; Zhang *et al.*, 2017).

The network design in terms of the physical flows, especially for thermal energy exchange, is bounded by the energy carrying streams’ properties. Energy exchange and, in particular, waste heat utilization, is often directly linked to geographical proximity, because possible heat losses restrain economic viability. Waste heat utilization refers to identifying processes producing excess heat (sources) and processes demanding heat (sinks) and utilize the energy carrying streams to achieve efficient heat recovery. Therefore, the strategy usually encountered in EIPs involves a centralized utilities system and a shared utilities network, in the form of a pipeline. The required data for network design depend on the type of optimization desired and, usually, for energy carrying streams, data such as temperature, flow rate, pressure, and enthalpy are essential. A knowledge-based system for EIP energy management is proposed by Zhang *et al.* (2017), which attempts to identify and match sources and sinks of waste heat through an ontology-based approach, utilizing data retrieved by the sensor network of each plant of the park, thus increasing knowledge interoperability and enhancing energy efficiency of an EIP. Contrary to waste heat, electricity is a type of energy that can be stored, for example in a battery or hydrogen storage, and then exchanged or reused ( Benedict *et al.*, 2018; Lawal *et al.*, 2021; Lütje *et al.*, 2019; Yeo *et al.*, 2019b; Zhang *et al.*, 2017). In the case of material flows, the factor influencing the network design is mass rate and for water flows the factors influencing the network design is flow rate and concentration of chemical components ( Yeo *et al.*, 2019b).

Moreover, the incorporation of PI techniques in ICT tools can facilitate the accurate identification of sources and sinks of industrial processes in a more sophisticated way. The combination of these methods enables the mapping of manufacturing procedures, the monitoring of process efficiency, the detection of ‘strengths’ and ‘weaknesses’ of the industrial processes and the identification of optimization and symbiotic opportunities. In other words, this is an all-inclusive approach where a user can discover the inefficiencies of a process unit or a whole production system, map the value chains and simultaneously is able to optimize the network by collaborating with other participants. One proposed method identified in this research is Multi-Layer Stream Mapping (MSM), a novel process efficiency methodology designed to identify symbiotic firm opportunities. Through this method, material and heat losses are identified and quantified more efficiently, thus enabling intra- or inter *-*firm exploitation of resources ( Holgado *et al.*, 2018).

## Discussion

This SLR attempted to gain an insight into the existing literature regarding IS platforms and important data points that influence the identification of synergistic opportunities and the implementation of IS through a digital tool. The findings were classified and discussed according to the categorization introduced by van Capelleveen *et al.* (2018a) regarding the type of information system facilitating IS. The systematic analysis uncovered two main types of important data points within an IS platform. The first type, ‘data required for synergy identification’, refers to the required data for accurate identification and matchmaking of the potential synergistic opportunities and includes six categories: i) general data, ii) inflow-outflow data, iii) economic data, iv) sharing practices data, v) internal practices data, and vi) supplementary data. The second type, ‘platform related information’, concerns key information regarding the platform and involves five different categories, namely: i) information related to the accessibility and openness of the platform, ii) sociability, iii) database used by the platform, iv) interactive visualization, and v) decision support the platform provides to the users through assessment methods.

The examined academic and grey literature documents were obtained utilizing the SLR PRISMA technique. Although the SLR technique was applied in order to ascertain credibility and systematic collection and organization of captured data, limitations of this study can be identified. This review analyzed the yielded literature, which is associated with the query search in the selected databases. Therefore, a constraint occurs, since the outcome reflects on the analyzed literature. It should be noted that the importance of data points is based on the quantity of the findings, thus conclusions on the significance of data points are based on the number of the included studies mentioning them. Moreover, the authors’ subjectiveness, although not fully eliminated, was reduced by two reviewers remaining independent during the literature’s screening process. Nevertheless, the assessment of eligibility of studies through full-text reading was conducted by one reviewer reporting to the supervising reviewer, as well as the identification and classification of findings, thus possible risk of personal bias exists.

It is evident from the research results that disclosure of several types of data is required for effective matching and overall, for the successful promotion of IS through digital tools. However, possible issues might arise concerning the willingness of the users to disclose their company’s sensitive and non-sensitive information. Important data is more likely to be shared if the actor understands the IS concept, is aware of the potential benefits resulting from IS and trusts the platform provider regarding confidentiality issues. A preliminary estimation of the stakeholders’ level of familiarity with the concept of IS and readiness to collaborate with other members of the network aims at understanding the maturity of the network and the expected extent of information disclosure. One proposed means to promote the platform is to demonstrate the potential benefits of a partnership, or historic case studies and their corresponding benefits. The role of the platform provider is crucial concerning confidentiality assurance, as users need to ensure that company’s valuable information will not be mishandled. Moreover, possible participation fees charged to the users by the platform might hinder the promotion of IS ( Fraccascia & Yazan, 2018; Kosmol, 2019; Kosmol & Leyh, 2019).

Additionally, an issue concerning IS platforms is that, until now, their development has mainly focused on functionality, whereas other aspects have not received equal attention. Undoubtedly, technical feasibility of a proposed partnership is imperative for the realization of IS. However, other elements of an IS network are to be regarded as well for successful promotion. Considering that the objective of IS initiatives is to create a resilient network of long-term partnerships, human interaction should not be neglected. Therefore, the proposed concept of linking potential collaborators by relying on more than just technical feasibility has been gaining attention. Social connections, available internal assets, technical expertise and managerial perspective of the company are some of the other aspects considered. This is a promising approach for further development of IS platforms since it intends to address needs and requirements beyond the technical prospect.

Another challenge concerning the broad application of digital tools facilitating IS addresses the lack of a common data format. Each company uses different information systems, methods and techniques that generate different datasets and unstructured data, which is not easily collected and managed. Therefore, the necessity for a common data format and data management model emerges in order to increase data quality and assist the effective identification of opportunities. A promising method to avoid data format complications is the application of implicit knowledge-based recommenders, where suggested synergies do not rely on external databases and the quality of data. In addition, the connection and adaption of the IS platform into the company’s internal organizational information systems is expected to advance the utilization of available data and the platform’s effectiveness ( Halstenberg *et al.*, 2017; van Capelleveen *et al.*, 2018b).

Furthermore, another deterring factor regarding information provision is related to manual entry of historical data, a cost-intensive and time-consuming procedure. Real-time data gathering techniques via sensors during manufacturing processes have been proposed, as they reflect valid and up-to-date information about the resources. In particular, the development of Internet of Things (IoT) monitoring and controlling technologies integrated in industrial processes related to streams’ characteristics could enhance waste identification and characterization, as well as real-time data gathering. Consequently, the utilization of possible online monitoring technologies in the industrial processes and data gathering techniques are useful for data sharing and management, especially in platforms facilitating EIPs ( Benedict *et al.*, 2018; Holgado *et al.*, 2019; Kosmol & Leyh, 2020; Lütje *et al.*, 2018; Zhang *et al.*, 2017).

With respect to the environmental impact assessment, based on the examined literature, only the evaluation of direct environmental benefits was described in detail, whereas more extensive approaches were not explicitly described. One main reason hindering the widespread adoption of more exhaustive methodologies estimating indirect environmental benefits is the level of complexity due to the large number of exchanges observed in IS networks. The complex nature of IS requires several assumptions, which cause deviation in the results. Therefore, a predefined set of rules are to be assigned in order to eliminate such deviations, including; the involved sectors, the baseline scenario for the environmental performance of the IS scenario, the selected system’s boundaries, the environmental indicators taken into consideration and other assumptions imposed to simplify the calculation of impacts. Moreover, data availability, disclosure or quality are some issues that determine the validity of the whole assessment. Therefore, a more detailed assessment method, even though it might be more inclusive, if the relevant stakeholder does not provide accurate data, it is not valid and transparent ( Haq *et al.*, 2021).

Overall, digital tools have been increasingly gaining attention for the facilitation of IS. Their role is especially important, as they can support information and knowledge exchange and utilize the obtained data for identification of potential synergies. However, it is considered that their full potential is yet to be discovered. Until now, many tools have been used but few have remained operational. Scholars deem that further research and advancement of IS platforms is required, to cover the full spectrum of aspects influencing IS initiatives. An important field that requires further research is the development of universal external databases that contain generic data, which utterly determine the recommendations of IS partnerships.

## Data availability

### Underlying data

All data underlying the results are available as part of the article and no additional source data are required.

### Reporting guidelines

Open Science Framework: PRISMA checklist for “Industrial symbiosis platforms for synergy identification and their most important data points: a systematic review”

https://doi.org/10.17605/OSF.IO/M3P2X ( Akrivou *et al.*, 2021).

Data are available under the terms of the Creative Commons Attribution 4.0 International license (CC-BY 4.0).
