# Supplementary material for: Industrial symbiosis platforms for synergy identification and their most important data points: a systematic review
Source: Open Res Eur. 2021 Nov 1;1:101. Originally published 2021 Aug 31. [Version 2] doi: 10.12688/openreseurope.13893.2 (PMC10445876; doi:10.12688/openreseurope.13893.2)
Supplement: Peer review history

# Peer review: Reviewer response for version 2

**DOI:** 10.21956/openreseurope.15373.r27946

**Published:** 2021-11-09

**Authors:** Athanasios Angelis-Dimakis

**Affiliations:** 1 Department of Chemical Sciences, School of Applied Sciences, University of Huddersfield, Huddersfield, UK

All comments have been addressed.

Are the rationale for, and objectives of, the Systematic Review clearly stated?

Yes

Is the statistical analysis and its interpretation appropriate?

Not applicable

Are sufficient details of the methods and analysis provided to allow replication by others?

Yes

Are the conclusions drawn adequately supported by the results presented in the review?

Yes

Reviewer Expertise:

Chemical Engineering, Industrial Symbiosis

I confirm that I have read this submission and believe that I have an appropriate level of expertise to confirm that it is of an acceptable scientific standard.

# Peer review: Reviewer response for version 1

**DOI:** 10.21956/openreseurope.14977.r27649

**Published:** 2021-10-15

**Authors:** Luca Fraccascia

**Affiliations:** 1 Department of Computer, Control, and Management Engineering “Antonio Ruberti”, Sapienza University of Rome, Rome, Italy

This paper develops a literature review aimed at highlighting the key data point of an industrial symbiosis platform. The topic is relevant as, although industrial symbiosis platforms are claimed to be a useful tool to (further) promote the development of industrial symbiosis, a limited effort has been devoted by the literature towards the development of such platforms.

 The methodology followed for the analysis is consistent with the aim of the article and rightly implemented. Concerning the exclusion criteria, I have two comments: (1) why the papers whose publication date is before 2016 have been excluded *a priori* is not justified and (2) non-peer-reviewed documents could have been included in the review, since this paper addresses a research field that is not well-established yet. Nevertheless, these two raised issues do not affect the validity of the outcome of the paper.

 From the technical perspective, the analysis of the data required for synergy identification and platform-related information looks accurate. I would suggest to add a table aimed at displaying all the data required for synergy identification and the platform-related information synoptically. This would enhance the sake of clarity.

 In the discussion, I appreciate the reference to non-technical issues (e.g., the willingness to share data), which could have been not properly addressed by the literature so far and, in my opinion, are a matter for future research. In my opinion, the discussion could have been further developed towards building a theoretical framework depicting the main features the “best” industrial symbiosis platforms should have, as well as discussing the implications of each feature on the platform's functionality and effectiveness. I suggest the authors develop such a framework, which can be useful to both academics and practitioners.

Are the rationale for, and objectives of, the Systematic Review clearly stated?

Yes

Is the statistical analysis and its interpretation appropriate?

Not applicable

Are sufficient details of the methods and analysis provided to allow replication by others?

Yes

Are the conclusions drawn adequately supported by the results presented in the review?

Partly

Reviewer Expertise:

Industrial symbiosis, circular economy

I confirm that I have read this submission and believe that I have an appropriate level of expertise to confirm that it is of an acceptable scientific standard.

# Response

**Published:** 2021-10-26

**Authors:** Chrysanthi Akrivou

Dear reviewer,

 Thank you very much for your time and effort - the revised version of this work has benefited significantly from your comments, as well as from other reviewers’ suggestions. Based on those recommendations, we have added some references and provided additional visualization of the results by including a table summarizing all the data required for synergy identification and platform-related information. Moreover, we have attempted to investigate the progress in the field, by comparing the results of this review to previously performed literature reviews (Grant *et al.*, 2010).

 In the following, we would like to respond to your individual comments:

*“Concerning the exclusion criteria, I have two comments: (1) why the papers whose publication date is **before 2016** have been excluded a priori is not justified and (2) **non-peer-reviewed** documents could have been included in the review, since this paper addresses a research field that is not well-established yet. Nevertheless, these two raised issues do not affect the validity of the outcome of the paper.”*

1. Addressed in the new version of the article in the literature research section.
2. Grey literature was also included in the research in order to involve also studies and projects that do not necessarily appertain to scientific background. For the scientific literature, peer-reviewed papers were selected in order to ensure the validity of their findings and statements.

*“I would suggest to add a table aimed at displaying all the data required for synergy identification and the platform-related information synoptically. This would enhance the sake of clarity.”*

 Addressed in the new version of the article in the results section (Table 5).

*“ In my opinion, the discussion could have been further developed towards building a theoretical framework depicting the main features the “best” industrial symbiosis platforms should have, as well as discussing the implications of each feature on the platform's functionality and effectiveness. I suggest the authors develop such a framework, which can be useful to both academics and practitioners.”*

 Theoretical framework, although indeed potentially very useful, would probably require a significant effort from the authors. However, the idea was added within the discussion section, included in the paragraph describing further research. Additionally, some findings that are considered meaningful to be shared as advice for academics and practitioners are shared in a newly added dedicated paragraph in the discussion section.

# Peer review: Reviewer response for version 1

**DOI:** 10.21956/openreseurope.14977.r27503

**Published:** 2021-10-07

**Authors:** Athanasios Angelis-Dimakis

**Affiliations:** 1 Department of Chemical Sciences, School of Applied Sciences, University of Huddersfield, Huddersfield, UK

Dear Authors,

 The submitted paper presented a systematic review of different existing industrial symbiosis platforms. The topic is interesting since the role of a facilitator is critical for the development of industrial symbiosis schemes.

 I am not sure I agree with the term "data points". With the term "data point", we usually refer to a discrete unit of information, a measurement. Do you mean that or is "type of data" (or something similar)?

 In terms or pre-existing work, Grant *et al *(2010) have previously studied the same topic, performing a very thorough literature review for the earlier attempts of IS ICT tools. Since your work spans between 2016-2021, it would be interesting to compare the two studies, in terms of approach and findings. You could have mentioned some of the characteristics Grant *et al.* (2010) have used (e.g. they have categorized the tools based on the step of of industrial symbiosis these refer to). Also it would be interesting to compare the two reviews and their findings.

 In terms of your analysis, the "type of exchange" classification is not clear to me. Why water is a separate category and not just a liquid material? How do you define materials?

 Why did you pick the year 2016 as the cut-off year for your analysis?

 I have added a few papers below that also seem relevant (one of them is dated July 2015, so you might want to ignore it, based on your cut-off decisions).

 The BeCircle platform developed as part of a Climate-KIC project (https://www.climate-kic.org/success-stories/be-circle/) could have been also mentioned, although I am not sure if the deliverable is public. If it is not, then you can ignore it based on your cut-off restrictions.

 Other than that, I think the review is thorough and extensive and properly analyzed and discussed.

Are the rationale for, and objectives of, the Systematic Review clearly stated?

Yes

Is the statistical analysis and its interpretation appropriate?

Not applicable

Are sufficient details of the methods and analysis provided to allow replication by others?

Yes

Are the conclusions drawn adequately supported by the results presented in the review?

Yes

Reviewer Expertise:

Chemical Engineering, Industrial Symbiosis

I confirm that I have read this submission and believe that I have an appropriate level of expertise to confirm that it is of an acceptable scientific standard, however I have significant reservations, as outlined above.

## References

[1] : Information and Communication Technology for Industrial Symbiosis. *Journal of Industrial Ecology* .2010;14(5) : 10.1111/j.1530-9290.2010.00273.x 740-753 10.1111/j.1530-9290.2010.00273.x

[2] : Development of the Tool SymbioSyS to Support the Transition Towards a Circular Economy Based on Industrial Symbiosis Strategies. *Waste and Biomass Valorization* .2017;8(5) : 10.1007/s12649-016-9748-1 1521-1530 10.1007/s12649-016-9748-1

[3] : Collaboration Platform for Enabling Industrial Symbiosis: Application of the By-product Exchange Network Model. *Procedia CIRP* .2017;61: 10.1016/j.procir.2016.11.225 263-268 10.1016/j.procir.2016.11.225

[4] : e-Symbiosis: technology-enabled support for Industrial Symbiosis targeting Small and Medium Enterprises and innovation. *Journal of Cleaner Production* .2015;98: 10.1016/j.jclepro.2014.08.051 336-352 10.1016/j.jclepro.2014.08.051

[5] : SWAN platform: A web-based tool to support the development of industrial solid waste reuse business models. *Waste Manag Res* .2021;39(3) : 10.1177/0734242X21989413 489-498 10.1177/0734242X21989413 33570022PMC7924101

# Response

**Published:** 2021-10-26

**Authors:** Chrysanthi Akrivou

Dear reviewer,

 Thank you very much for your time and effort - the revised version of this work has benefited significantly from your comments, as well as from other reviewers’ suggestions. Based on those recommendations, we have added some references and provided additional visualization of the results by including a table summarizing all the data required for synergy identification and platform-related information. Moreover, we have attempted to investigate the progress in the field, by comparing the results of this review to previously performed literature reviews (Grant *et al.*, 2010).

 In the following, we would like to respond to your individual comments:

*"I am not sure I agree with the term "data points". With the term "data point", we usually refer to a discrete unit of information, a measurement. Do you mean that or is "type of data" (or something similar)?"*

 The authors have investigated this topic carefully during several stages of the systematic review preparation.  Our approach is based on the perception that, any fact or piece of information is considered a data point. In data analysis and statistics, the term “data point” is used to describe a piece of information that describes one unit of observation or one object (Frank & Todeschini, 1994). In that sense any single fact is seen as a data point, as the term is used as something that defines a certain feature of the examined topic, as in (our) paper. In this paper, the term is not used in an analytical context, in which a data point is usually derived from a single or a set of experimental measurements and can be represented numerically and/or graphically (Montag, Duke, & Markowetz, 2016). Moreover, the term “type of data” appertains to the data points being either words, numbers, or other symbols, in order to be queried from data tables. Therefore, in this research, the term is used as a set of observations that imply the same feature of an IS platform, and what we search for is the observations that guide towards a certain feature or functionality of the platform.

*"In terms or pre-existing work, Grant et al (2010) have previously studied the same topic, performing a very thorough literature review for the earlier attempts of IS ICT tools. Since your work spans between 2016-2021, it would be interesting to compare the two studies, in terms of approach and findings. You could have mentioned some of the characteristics Grant et al. (2010) have used (e.g. they have categorized the tools based on the step of industrial symbiosis these refer to). Also, it would be interesting to compare the two reviews and their findings."*

 Addressed in the new version of the article, in the Introduction and in the Discussion sections.

*"In terms of your analysis, the "type of exchange" classification is not clear to me. Why water is a separate category and not just a liquid material? How do you define materials?"*

 The reason the "type of exchange" classification was structured as such (water, material, energy) was that previous literature discussed intercompany exchanges using that approach (Yeo *et al*. 2019b, Lawal *et al*. 2020, Bakogianni *et al*., 2019). For example, the Online Brine platform (Bakogianni et al., 2019) facilitates intercompany exchanges of heat (energy), water and nutrients (materials). Moreover, one of the objectives of this classification was to detect, if digital tools supporting solely water related exchanges exist.

*"Why did you pick the year 2016 as the cut-off year for your analysis?"*

 Addressed in the new version of the article in the literature research section.

*"The BeCircle platform developed as part of a Climate-KIC project (https://www.climate-kic.org/success-stories/be-circle/) could have been also mentioned, although I am not sure if the deliverable is public. If it is not, then you can ignore it based on your cut-off restrictions."*

 Seems like the deliverables are not available, therefore can be disregarded based on the cut-off restrictions.

*"I have added a few papers below that also seem relevant (one of them is dated July 2015, so you might want to ignore it, based on your cut-off decisions)."*

 About the proposed literature, Grant *et al*., 2010 is included (addressed in the article as a reference) in the introduction and the Discussion section. Angelis-Dimakis *et al*., 2021, Álvarez *et al*., 2017, Raabe *et al*., 2017 was added based on suggestions, since it is considered relevant to the topic analysed by this review, in the Introduction and the Discussion section. Cecelja *et al*., 2015 is a paper out of the time span of our review, therefore will not be included.

 REFERENCES:  Bakogianni D, Skourtanioti E, Meimaris D, *et al.*: Online Brine platform: a tool for enabling industrial symbiosis in saline wastewater management domain. *2019 15th International Conference on Distributed Computing in Sensor Systems (DCOSS)*. 2019; 430–435.    Frank , I., & Todeschini, R. (1994). *The Data Analysis Handbook.* Elsevier. Lawal M, Wan Alwi SR, Manan ZA, *et al.*: Industrial symbiosis tools—A review. *J Clean Prod.* Elsevier Ltd, 2021; **280**(Part 1): 124327. Montag, C., Duke, É., & Markowetz, A. (2016). Toward Psychoinformatics: Computer Science Meets Psychology. *Computational and Mathematical Methods in Medicine, vol. 2016*, 10. doi:https://doi.org/10.1155/2016/2983685 Yeo Z, Masi D, Low JSC, *et al.*: Tools for promoting industrial symbiosis: A systematic review. *J Ind Ecol.* 2019b; **23**(5): 1087–1108.
